# Alectinib treatment improves photodynamic therapy in cancer cell lines of different origin

**DOI:** 10.1186/s12885-021-08667-x

**Published:** 2021-08-30

**Authors:** Bernhard Gillissen, Antje Richter, Frank Essmann, Wolfgang Kemmner

**Affiliations:** 1 Department of Hematology, Oncology, and Tumor Immunology, Charité - University Medicine Berlin, Campus Berlin-Buch, Humboldt University, Berlin, Germany; 2 Dr. Margarete-Fischer-Bosch Institute of Clinical Pharmacology and University of Tuebingen, Stuttgart, Germany, Auerbachstr. 112, 70376 Stuttgart, Germany; 3 Research Group Translational Oncology, Experimental and Clinical Research Center at the Max- Delbrueck-Center for Molecular Medicine, Lindenberger Weg 80, 13125 Berlin, Germany

**Keywords:** Photodynamic therapy, Protoporphyrin-IX, Ferrochelatase, Alectinib, Gastrointestinal carcinomas

## Abstract

**Background:**

Photodynamic therapy with a photosensitizer such as protoporphyrin-IX, a light sensitive metabolite of heme synthesis, is a highly selective treatment for various carcinomas. In previous studies, we found a significant down regulation of the relevant enzyme ferrochelatase in gastrointestinal carcinomas leading to an accumulation of protoporphyrin-IX within the tumor cells. Recent studies showed that a novel anti-cancer drug, Alectinib, an orally available, highly selective, potent second-generation inhibitor of anaplastic lymphoma tyrosinkinase binds to ferrochelatase. Therefore, we were interested to see whether Alectinib treatment might lead to an accumulation of protoporphyrin IX.

**Methods:**

Tumor cells of different origin were cultured, treated with LED-light and Alectinib. Results were gained by flow cytometry, immunohistochemistry and western blotting. Apoptosis was determined by flow cytometric analysis of Annexin V-FITC stained cells. In addition, cells were counterstained with propidium iodide to distinguish early apoptotic cells and late apoptotic/necrotic cells.

**Results:**

Here, we report that photodynamic treatment of tumor cell lines of different origin in combination with Alectinib increased protoporphyrin-IX specific fluorescence and concomitantly cell death.

**Conclusions:**

The usage of Alectinib could be another step for enhancing the effectiveness of photodynamic therapy. Further experiments will show whether photodynamic therapy in combination with Alectinib could be a new strategy for the treatment of e.g. peritoneal disseminated carcinomas.

**Supplementary Information:**

The online version contains supplementary material available at 10.1186/s12885-021-08667-x.

## Background

Photodynamic therapy (PDT) is a promising alternative approach for improving cancer treatment. This approach has several advantages such as noninvasive treatment, repeatability without side effects, little or no scar after healing and short treatment times. However, treatment efficacy depends on accurate light delivery to the tumor and sufficient tissue oxygenation. Crucial for photodynamic therapy is the choice of a photosensitizer such as protoporphyrin IX (PpIX), a light sensitive metabolite of heme synthesis. After treatment with light, PpIX is able to transfer that energy to molecular oxygen, leading to generation of reactive oxygen species (ROS) responsible for cellular oxidation and subsequent necrotic and/or apoptotic cell death of tumor cells [1]. PpIX is under extensive study as a photosensitizer for tumor detection and photodynamic therapy [2]. On the one hand, generation of PpIX depends on a sufficient intracellular delivery of the precursor substance 5-aminolevulinic acid (5-ALA). On the other hand, generation of PpIX can be increased by down-regulation of the activity of the ferrochelatase FECH (EC 4.99.1.1), since PpIX is converted into heme by insertion of iron by FECH localized within the inner mitochondrial membrane.

In previous studies, we were able to demonstrate a significant down regulation of FECH in gastrointestinal carcinomas leading to the accumulation of PpIX within the tumor cells [3]. Later, we introduced the use of a siRNA-based probe that is capable of amplifying the specific endogenous fluorescence emission of PpIX within cancerous tissue by inhibition of FECH expression [4]. By measuring the thermal stability of proteins ligand binding in living cells, FECH was identified as a target of several kinase inhibitors in recent studies [5, 6]. One of them, Alectinib (CH5424802), is an orally available, highly selective, potent second-generation inhibitor of anaplastic lymphoma tyrosinkinase (ALK). These results suggest that Alectinib is able to block the activity of FECH. Alectinib demonstrated a favorable safety profile and clinically meaningful response in patients with ALK-positive metastatic NSCLC and was granted accelerated approval by the United States Food and Drug Administration (FDA) on December 11, 2015 [7]. Alectinib has shown strong efficacy in the treatment of ALK-positive non-small-cell lung cancer [8].

The aim of this study was to find out whether pretreatment of tumor cells with Alectinib leads to an enhanced concentration of PpIX due to inhibition of FECH and how this affects the induction of cell death. We focused on induction of PpIX in the breast cancer cell line MDA-MB-231. First, we tried to determine quantitatively the effect of addition of 5-ALA (section II) and light treatment (section III) on PpIX accumulation and cell death (section IV). Then we used this data for assessment of the effect of Alectinib on PpIX accumulation in cancer cell lines of different origin. Here, we report that treatment of tumor cells with Alectinib increased PpIX-fluorescence and concomitantly cell death of tumor cells after photodynamic treatment. Thus, the usage of Alectinib could be another step for improving photodynamic therapy of various tumor entities.

## Methods

### Chemicals and reagents

BSA, 5-aminolevulinic acid (ALA), DMSO, and 4′,6-Diamidino-2-phenylindole dihydrochloride hydrate (DAPI) were purchased from Sigma-Aldrich (Munich, Germany); RPMI cell culture medium, Dulbecco’s Modified Eagle’s Medium, fetal calf serum, phosphate buffered saline, antibiotics, Trypsin/EDTA and L-Glutamine from GIBCO (Eggenstein, Germany), and Accutase form BIOZOL (Eching, Germany). Alectinib and Crizotinib were bought from Absource Diagnostics GmbH (Munich, Germany) solved in DMSO (1 mM stock solution) and stored at − 20 **°**C. Annexin V-FITC Apoptosis Detection KIT was obtained from BD-Biosciences (Heidelberg, Germany). Fluorescent mounting antifading reagent was obtained from (DAKO, CA, USA).

### LED light source

A special light device has been constructed to allow illumination with light of defined spectral composition and calibrated intensity. A Light Emitting Diode (LED) UV5TZ-405-30 (Bivar Inc., Irvine, CA) was placed into the center of a 30 mm concave reflector. The LED emits light at a center wavelength of 405 nm. The half-width Δλ of the spectral profile is 35 nm. Illumination intensity (power per area) was measured with a “Solo 2” Laser Power and Energy Meter (Gentec-EO, Quebec, Canada) in the center of the illuminated area and at different distances (Supplemental Fig. 4). A desired intensity was achieved by adjustment of the supply current. Within experiments, the LED lamp was directly positioned 25 mm above the tissue culture 6 well plate for cell incubation (Sarstedt AG & Co., Nümbrecht, Germany), with an intensity of 4 mW/cm2.

### Cell culture

Human breast cancer cell lines MDA-MB-231 (**ACC 732,** Deutsche Sammlung für Mikroorganismen und Zellkulturen DSMZ, Braunschweig, Germany) and colorectal carcinoma cells SW480 (CCL-228, ATCC, Manassas, VA, USA) were maintained in RPMI (PAA Laboratories, Pasching, Austria) supplemented with 10% FCS (PAA Laboratories), 2 mM Glutamine. Gastric carcinoma cells MKN28 (JCRB0253, JCRB Cell Bank, Tokyo, Japan), and NSCLC cancer cell lines NCI-H460 (HTB-177, ATCC) and NCI-H1299 (CRL-5803, ATCC) were kept in DMEM low Glucose (Invitrogen, Karlsruhe Germany), 10% FCS, 2 mM Glutamine. Esophageal adenocarcinoma cells OE33 (ACC 706, DSMZ) were maintained in DMEM 1:1 mixture of low and high Glucose, 10% FCS, 2 mM Glutamine. MCF-10A (CRL-10317, ATCC) cells were grown in DMEM/F12 (1:1), PAN-Biotech, P04–41250 (L-Glutamine (2,5 mM); HEPES (15 mM), NaHCO3 (1,2 g/L), Na-Pyruvat (1 mM)) supplemented with 10% FCS, hEGF (20 ng/ml) Hydrocortison (0,5 μg/ml) Insulin (10 μg/ml) and cholera toxin (100 ng/mL). HCT116 wild-type cells and the isogenic double knockout subline HCT116-Bax^−/−^/Bak^−/−^ were kindly provided by Dr. R. J. Youle, NIH (Bethesda, MD) [9]. All cells were grown in DMEM supplemented with 10% FCS, 100,000 units/liter penicillin, and 0.1 g/liter streptomycin at 37 °C with 5% CO_2_ in a humidified atmosphere. Media and culture reagents were from Invitrogen unless stated otherwise. Upon reaching approximately 85% confluence, cells were detached with Accutase (Sigma-Aldrich, Germany) in PBS containing 0.5 mM EDTA, sub cultured in new flasks and used for the assays.

### Cell treatment procedures

Cancer cells were seeded into 6-well plates (2 × 10^5^ cells/2 ml). The next day, in some of the experiments, cells were treated with Alectinib for 24 h at 37 °C in a cell incubator. Then, cells were treated with ALA for 3 h and, depending on the experimental setting, with 10 μM of the pan caspase inhibitor Q-VD-OPh. After washing, cells were exposed to a LED emitting light at a wavelength of 405 nm. Subsequently, cells were kept for 18 h at 37 °C in a cell incubator. Apoptosis was determined using an Annexin V-FITC detection kit (BD Biosciences) according to the manufacturer’s instructions. For flow cytometric analysis, at least 10,000 cells were evaluated using a FACSCalibur or LSRFORTESSA (BD Biosciences). Cell cycle distribution and sub-G1 fraction were determined in a quantitative way using the CellQUEST™ program. Annexin V positive-PI negative cells are counted as early apoptotic cells. All experiments were done at least in triplicates.

### Immunoblotting

After trypsination, cells were washed twice with ice-cold PBS and lysed in 10 mM Tris-HCl pH 7.5, 137 mM NaCl, 1% Triton X-100, 2 mM EDTA, 1 mM pepstatin, 1 mM leupeptin, and 100 μM phenylmethyl sulfonylfluoride (PMSF). Protein concentration was determined using the Thermo Scientific Pierce BCA Protein Assay Kit (Life Technologies GmbH) and equal amounts of protein (20 μg per lane) were separated by SDS-PAGE. After electrophoresis proteins were transferred onto 0.2 μm nitrocellulose membranes by semi-dry blotting using a BioRad Trans-blot SD transfer cell. Membranes were blocked in blocking buffer (5% BSA, 0.1% Tween-20 in PBS) for 1 h, followed by an overnight incubation with primary antibodies in blocking buffer at 4 °C. Primary antibodies used were anti-Bak mAb (clone TC102) from Calbiochem (Merck KGaA, Darmstadt, Germany), anti-Bax mAb (clone YTH-2D2) from Trevigen (Gaithersburg, USA) and mouse anti-FECH (A-3) from Santa Cruz Biotechnology (Heidelberg, Germany). Secondary antibodies coupled to horseradish peroxidase (from Promega, Mannheim, Germany) and Pierce ECL Western Blotting Substrate reagents were used to detect proteins by chemoluminescence.

### Microscopy

Cancer cells were seeded into 6-well plates (2 × 10^5^ cells/2 ml), treated for 24 h with 10 μM Crizotinib or Alectinib. Thereafter, cells were incubated with 100 μM ALA for 4 h. Microscopy images were then taken using an Axiovert 200 inverse fluorescence microscope (Carl Zeiss, Inc.) equipped with a Hamamatsu ORCA-ER digital camera using the Openlab software (Improvision). Mean fluorescence intensity of images was analyzed using ImageJ 1.53a software (https://imagej.nih.gov/ij/).

#### Statistical analysis

Experimental data were presented as the mean ± standard deviation (SD). Statistical analyses were performed using the GraphPad Prism 5. Student t-test was used for data analysis between two groups. *P*-values of less than 0.05 were considered statistically significant.

## Results

I. **Alectinib induces PpIX-Fluorescence in tumor cells**

**Fig. 1:**
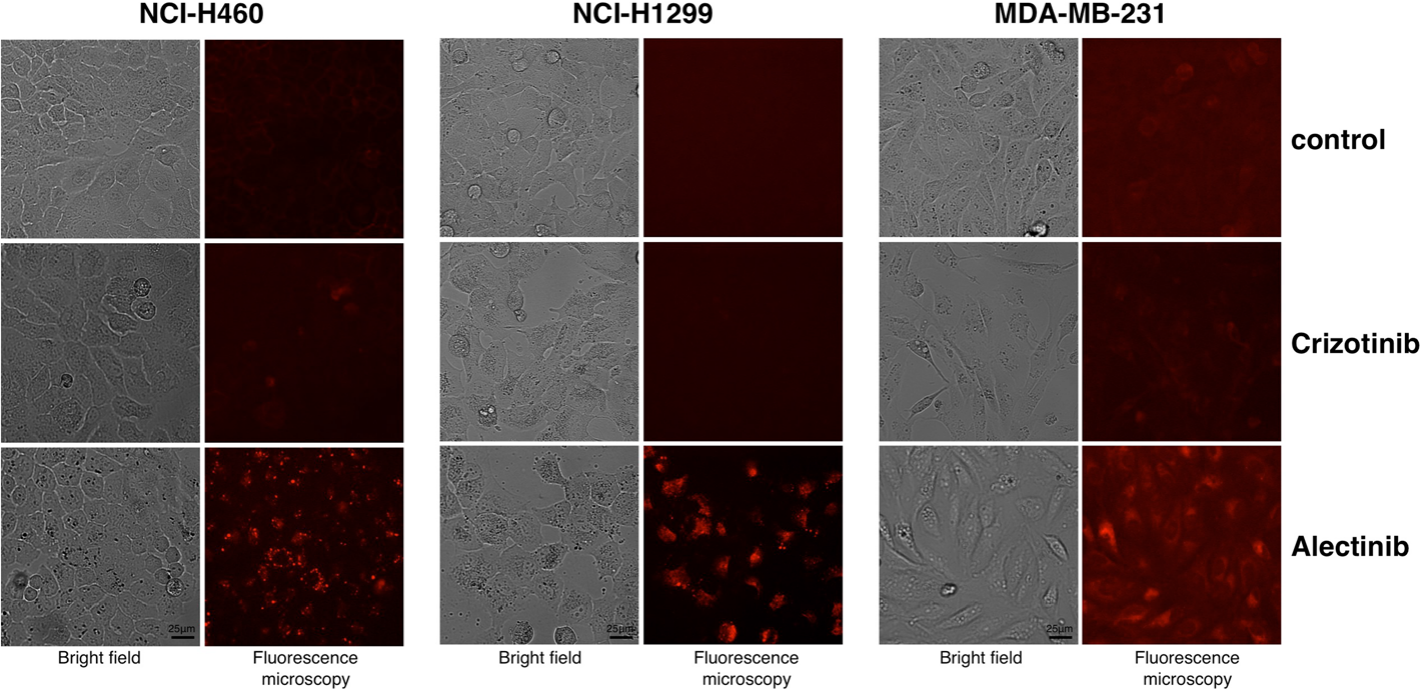
Fluorescence of NSCLC and breast cancer cells after treatment with Alectinib. NSCLC cells H460, H1299 and MDA-MB-231 breast cancer cells were treated for 24 h with 10 μM Crizotinib (middle rows) or Alectinib (lower rows) in 6 well plates. Thereafter, cells were incubated with 100 μM ALA for 4 h. Bright field microscopy pictures (left columns) and PpIX-Fluorescence (right columns) were assessed in parallel using Axiovert 200 M Inverted Fluorescence Microscope. Treatment with Alectinib induced PpIX-fluorescence in the cancer cells, while Crizotinib was not effective. (Magnification bar = 25 μm)

Recent studies showed that a novel anti-cancer drug, Alectinib, is able to bind the FECH-enzyme. Thus, we suspected that Alectinib inhibits the activity of FECH within the heme metabolism. In an initial experiment, we examined how the treatment of cancer cells with Alectinib affects the generation of PpIX by fluorescence microscopy detection. As a control treatment here, we examined Crizotinib which is another multiple tyrosine kinase inhibitor (TKI) of ALK and has also been approved for ALK-rearranged NSCLC [8]. Thus, the effects of both TKIs on generation of PpIX-fluorescence in human breast cancer cell line MDA-MB-231 were assessed. Since both TKIs have been approved for treatment of NSCL also their effects on PpIX accumulation in the ALK positive NSCLC tumor cell lines H1299 and H460 [10] were examined. Briefly, cells were seeded into 6-well plates (2 × 10^5^ cells/2 ml RPMI with 10% FCS, 1% Glutamine). Next day, cells were treated for 24 h at 37 °C in a cell incubator with 10 μM Alectinib or Crizotinib. Upon Alectinib or Crizotinib treatment, cells were incubated with 100 μM ALA/RPMI medium without phenol red for 3 h. Then, cells were transferred into an Axiovert 200 M Inverted Fluorescence Microscope equipped with a 545/30 nm (excitation), 620/60 nm (emission) filter set, and bright field microscopy pictures and PpIX-Fluorescence images were obtained by OpenLab software (Improvision). Treatment with 10 μM Alectinib induced PpIX-fluorescence in the two NSCLC cell lines as well as in MDA-MB-231 breast cancer cells, while Crizotinib was not effective (Fig. 1, mean fluorescence-intensity of three experiments are shown in supplemental Fig. 1). Similarly, control treatment with 1% DMSO in PBS was not effective. Thus, we were able to demonstrate that Alectinib in fact induces an accumulation of PpIX as detected by its specific fluorescence. These results are in accordance with data showing that only Alectinib binds to the FECH protein and not Crizotinib [5, 6]. Interestingly, Alectinib only acted upon assisting PpIX accumulation in the ALK positive NSCLC tumor cell lines H1299 and H460, however Crizotinib did not (Fig. 1).

II. **Addition of aminolevulinic acid for induction of PpIX-Fluorescence in tumor cells**

**Fig. 2:**
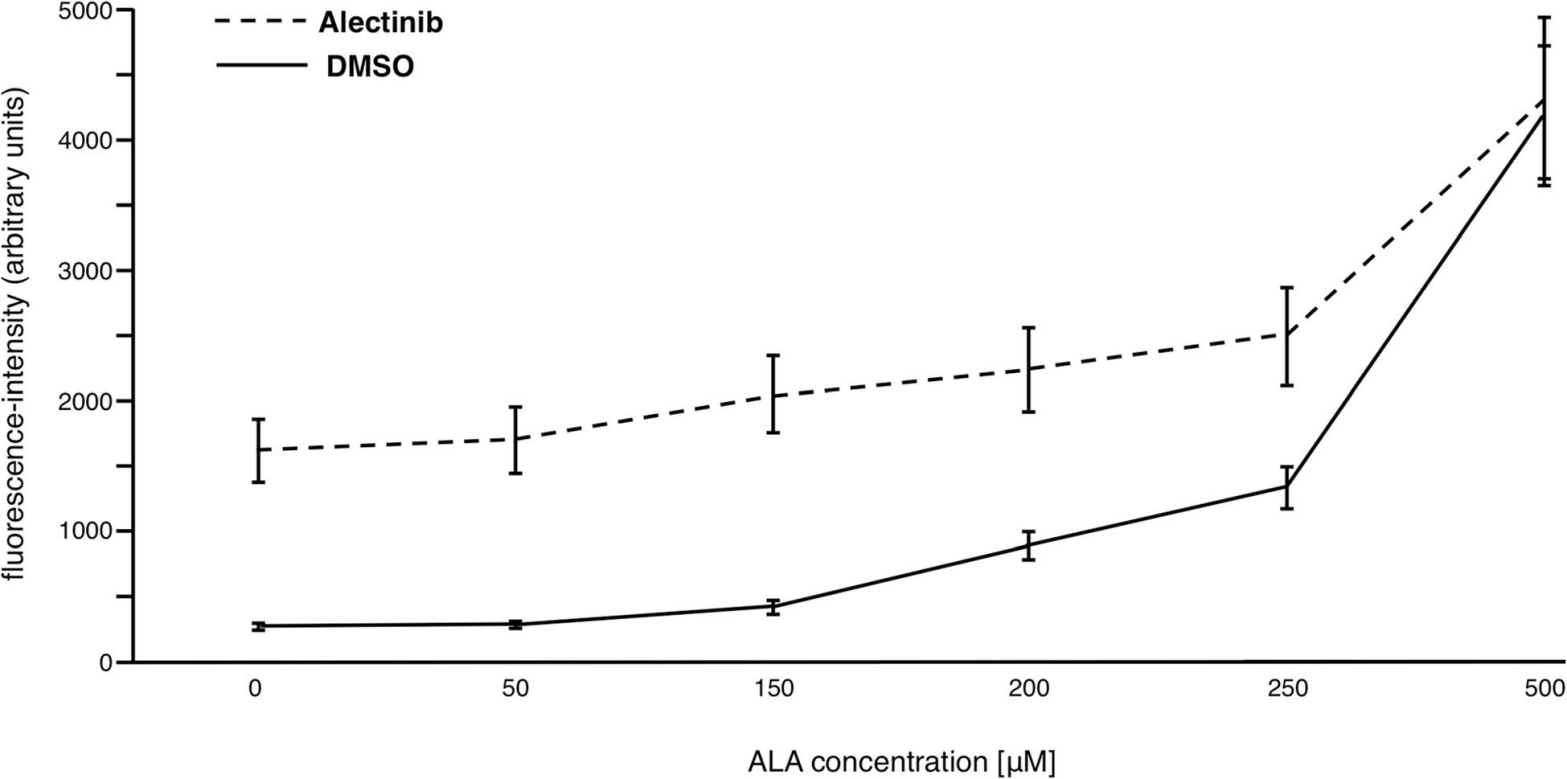
**Addition of ALA increased Alectinib-stimulated PpIX-fluorescence breast cancer cells**. Human breast cancer cell lines MDA-MB-231 were incubated with 10 μM Alectinib for 24 h. Subsequently, cells were treated with ALA (X-axis) for 3 h. PpIX-fluorescence (Y-axis, arbitrary units) of the cells was directly assessed by flow cytometry with an excitation of 405 nm and emission at 698 nm (Filter 695/40). Treatment with Alectinib (dashed line) dissolved in DMSO resulted in a higher PpIX-fluorescence compared to treatment with DMSO alone (solid line) for concentrations of ALA up to 250 μM. Depicted is the median of three measurements

The results of the initial experiment (I) encouraged us to study the effects of Alectinib in detail. On the one hand, generation of PpIX can be increased by down-regulation of the activity of FECH, on the other hand, generation of PpIX depends on a sufficient intracellular delivery of the precursor substance (5-ALA). Therefore, it was necessary to assess the effect of Alectinib on PpIX-fluorescence with regard to a treatment of cells with various concentrations of 5-ALA. To this end, the human breast cancer cell line MDA-MB-231 was incubated with 10 μM Alectinib in 1% DMSO for 24 h at 37 °C in a cell incubator. Since the generation of PpIX depends also on a sufficient intracellular concentration of the precursor 5-ALA, cells were pretreated with various concentrations of 5-ALA for 3 h. After another washing step, PpIX-fluorescence of the cells was directly assessed by flow cytometry with an excitation of 405 nm and emission at 698 nm (Filter 695/40). Treatment with Alectinib dissolved in DMSO resulted in a higher PpIX-fluorescence compared to treatment with DMSO alone for concentrations of 5-ALA up to 250 μM. In any case, there is a significant difference between treatment with Alectinib and control (*p* < 0.001, t-test), except at a 5-ALA-concentration of 500 μM. The highest significant difference between Alectinib and control was found at an ALA-concentration of 150 μM (Fig. 2). ALA exceeds the effect of Alectinib on PpIX-fluorescence at higher concentrations. For further experiments we chose a 5-ALA concentration of 150 μM.

III. **Induction of PpIX-fluorescence and cell apoptosis by treatment with light.**

**Fig. 3:**
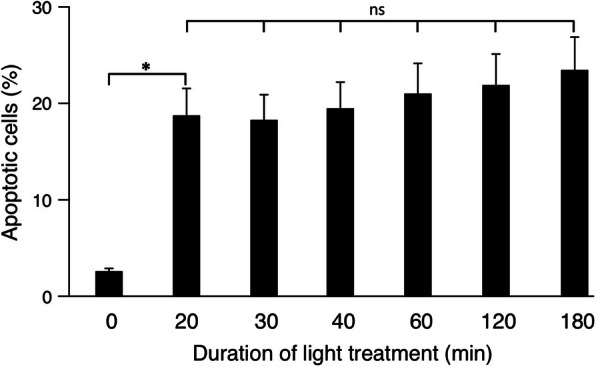
**Induction of phosphatidylserine exposure in breast cancer cells due to LED light treatment**. Light induction of apoptosis in MDA-MB-231 breast cancer cells pretreated with 250 μM ALA. LED lamp emitting light at a wavelength of 405 nm was operated at 25 mA leading to an intensity of 4 mW/cm^2^. Duration of light treatment was between 20 min. and 180 min. (X-axis). Apoptosis was determined using an Annexin V-FITC detection kit by flow cytometry. Exposure of ALA-pretreated MDA-MB-231 breast cancer cells to LED light led to a rate of about 20% apoptotic cells. (Data show mean ± SD of three independent experiments; * *p* < 0.05)

After treatment with light, PpIX is able to transfer that energy to molecular oxygen, leading to generation of reactive oxygen species (ROS) responsible for cellular oxidation and subsequent apoptosis or necrosis of tumor cells [1]. Here, we were interested to see how a specific light treatment induced apoptosis in MDA-MB-231 breast cancer cells. To this end, exposure of cells to LED light of 405 nm was assessed. Cells were pretreated with 250 μM 5-ALA for 3 h. After washing, cells were exposed to a LED emitting light at a wavelength of 405 nm, which was operated at 25 mA leading to an intensity of 4 mW/cm^2^. Duration of light treatment was between 20 min and 180 min within the cell incubator. After light treatment, apoptosis was determined by flow cytometric analysis of cells, stained for phosphatidylserine exposure by use of an Annexin V-FITC detection kit (BD Biosciences). Exposure of 5-ALA pretreated MDA-MB-231 breast cancer cells to LED light led to a rate of about 20% apoptotic, Annexin V-FITC positive cells (Fig. 3). A treatment of 60 min turned out to be sufficient for this apoptosis rate. There were no significant differences between any of the exposure times. Recently, it has shown that the triple-negative breast cancer cells MDA-MB-231 display a high susceptibility to PDT [11]. Since we want to compare the MDA-MB-231 cells with other cell lines which might be less susceptible an exposure time of 60 min was chosen for further experiments.

IV. **Induction of phosphatidylserine exposure by Alectinib and light treatment**

**Fig. 4:**
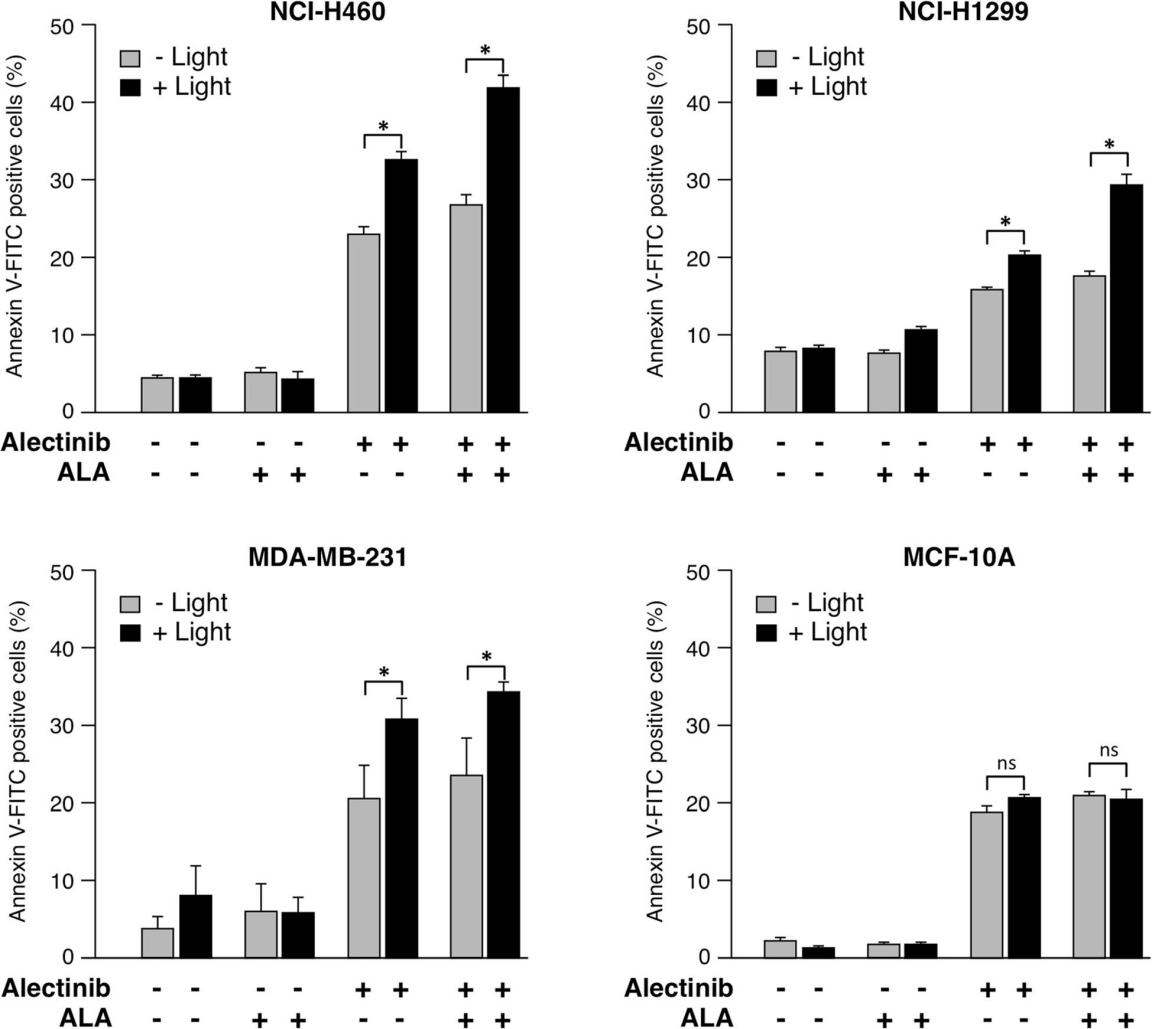
**Light treatment increased Alectinib-induced phosphatidylserine exposure of NSCLC and breast cancer cells**. NSCLC cells H460, H1299 and MDA-MB-231 breast cancer cells were / or were not treated with 10 μM Alectinib, 150 μM ALA, and exposure to a LED lamp emitting light at a wavelength of 405 nm (X-axis). Phosphatidylserine exposure (Y-axis) was determined using an Annexin V-FITC detection kit by flow cytometry. Light treatment (black columns) increased the effect of Alectinib (right side of each figure) significantly. In H460, H1299 and MDA-MB-231 cell lines light treatment (black columns) increased the effect of Alectinib (right side of each figure) significantly (* *p* < 0.05). In contrast, in the non-tumorigenic human breast cell line MCF-10A the rate of cell death is not increased by additional LED light irradiation. (mean ± SD of three independent experiments; * *p <* 0.05)

Now we asked how light treatment affects cell death induction by Alectinib. The breast cancer cell line MDA-MB-231 but also human NSCLC cancer cell lines NCI-H460 and NCI-H1299 were treated with 10 μM Alectinib as above (II). After washing, cells were treated with 150 μM 5-ALA for 3 h. After another washing step, cells were exposed to a LED emitting light as above (III) and apoptosis was determined by flow cytometric analysis of Annexin V-FITC stained cells. ALA-treatment did not significantly change induction of apoptosis. In contrast, additional Alectinib treatment induced phosphatidylserine exposure in all three human cancer cell lines, around 26% of the NCI-H460, 18% of NCI-H1299 and 26% of the MDA-MB-231 cells were positive for Annexin V-FITC staining (Fig. 4). Upon light exposure, however, cells showing a positive staining for Annexin V-FITC increased up to 42, 30 and 35%, respectively (Fig. 4, columns on the right side). In order to analyze whether treatment with Alectinib could be deleterious for the normal peripheral tissue in addition we assessed cell death induction by Ale/ALA/Light treatment in the non-tumorigenic human breast cell line MCF-10A. As shown for the other tumor cell lines, Alectinib induced cell death also in MCF-10A cells. However, the rate of cell death is not increased by additional LED light irradiation in MCF-10A cells (Fig. 4). In contrast, exposure of the cancer cell lines to LED light (405 nm) increased the effect of Alectinib significantly (*p* < 0.05) for all cell lines. Therefore, this combination of Alectinib treatment and exposure to a LED emitting light at a wavelength of 405 nm was used in further experiments.

V. **Induction of apoptosis by Alectinib in various cancer cell lines**

**Fig. 5:**
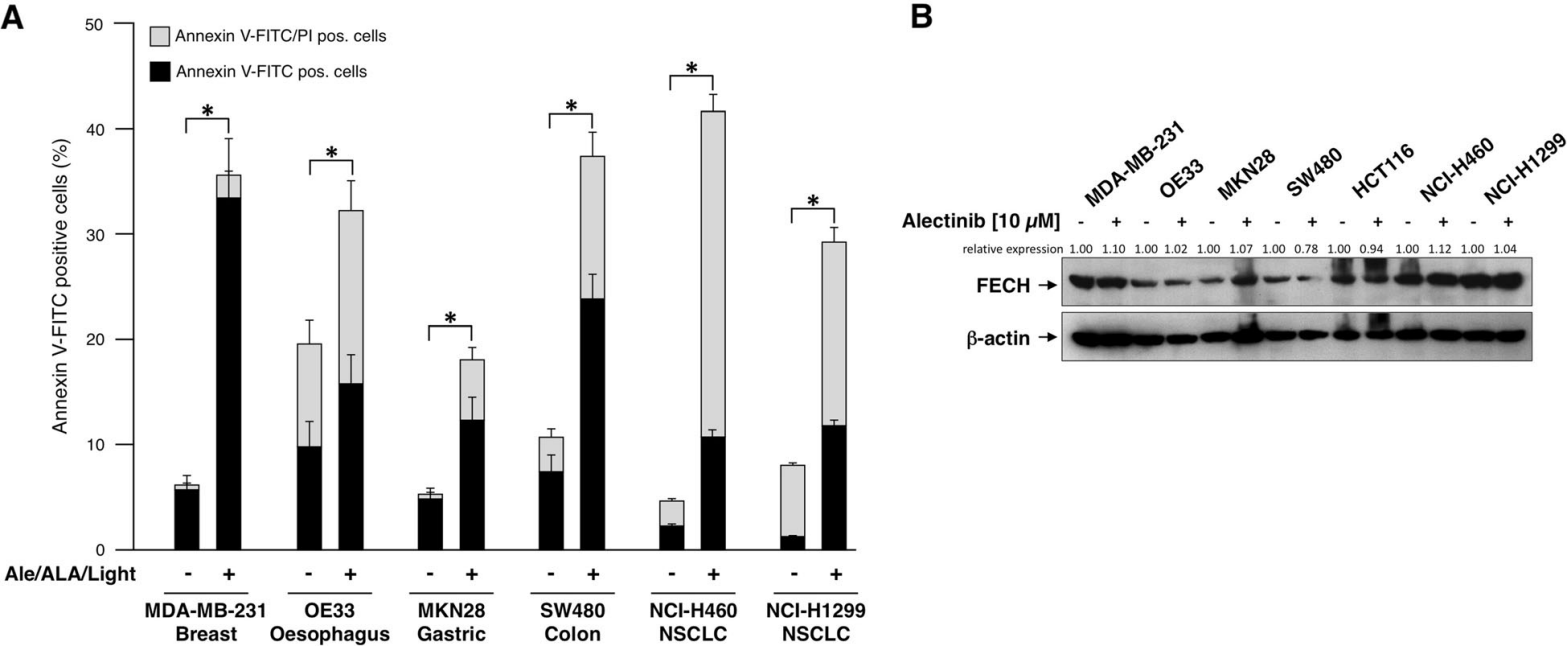
**Apoptosis induction by Alectinib and light treatment in carcinoma cell lines**. A) Cells of various cancer cell lines such as the breast carcinoma line MDA-MB-231, esophageal adenocarcinoma OE33, gastric carcinoma MKN28, colorectal carcinoma SW480 and NSCLC cells H460, H1299 were treated with DMSO alone or with 10 μM Alectinib/DMSO, 150 μM ALA, and LED light. Cells were stained using an Annexin V-FITC detection kit, counterstained by PI and apoptotic cells were determined by flow cytometry. Annexin V-positive/PI-negative cells were considered as early apoptotic cells, Annexin V-positive/PI-positive cells were late apoptotic/necrotic cells. Combined treatment of cells with Alectinib, ALA and LED light led to enhanced cell death in all the cell lines. (mean ± SD of three independent experiments; * *p <* 0.05) B) Densitometric analysis of Western Blot reveals that all cell lines strongly express FECH and that FECH expression levels were not changed by Alectinib treatment, except for SW480 cells where expression is slightly reduced. Level of FECH expression was normalized to respective actin control and presented as relative protein expression level in relation to respective wild type control. (Analysis was performed twice. Both WB including quantification and indication of the mean value are shown in the supplemental Fig. 2)

Cells of various cancer cell lines such as the breast carcinoma line MDA-MB-231, esophageal adenocarcinoma OE33, gastric carcinoma MKN28, colorectal carcinoma SW480, and NSCLCs NCI-H460 and NCI-H1299 were treated as above (10 μM Alectinib, 150 μM 5-ALA for 3 h, and LED emitting light at a wavelength of 405 nm for one hour). Apoptosis was determined by flow cytometric analysis of Annexin V-FITC stained cells. In addition, cells were counterstained with propidium iodide (PI) to distinguish early apoptotic cells (Annexin V-positive/PI-negative) and late apoptotic/necrotic cells. Combined treatment of cells with Alectinib and LED light led to enhanced cell death in all cells (Figs. 5 and 7). However, the proportion of early apoptotic cells and late apoptotic/necrotic cells varied between the different cell lines. Treatment with Alectinib and LED Light exposure enhances cell death in the cancer cell lines MDA-MB-231, MKN-28 and OE33 also without addition of ALA. In all four cell lines (including SW-480) cell death induced by LED Light is further stimulated by pre-treatment of cells with 5-ALA (Fig. 7). We next examined the status of FECH of all cell lines upon treatment with Alectinib by Western blotting using an antibody specific for FECH and the ImageLab software 6 (Bio-Rad) for quantification of the relative expression (Fig. 5, right). As expected all cell lines strongly express FECH and Alectinib treatment did not lead to a change in FECH expression, with the exception of SW480 cells where FECH expression was slightly reduced. Interestingly, FECH expression did not correlate with the sensitivity of the cells for cell death induction, indicating that kinetics of cell death induction by apoptotic or necrotic signaling pathways differ between cell lines. In addition to FECH we therefore examined expression of key pro- and anti-apoptotic Bcl-2 family members. All cell lines strongly express Bak and Bax, with the strongest Bax expression seen in MKN28 and SW480 cells (Supplemental Fig. 3). In comparison, the expression of the anti-apoptotic Bcl-2 and Bcl-x_L_, important inhibitors of Bax and Bak, respectively, varies more between cell lines. Bcl-2 expression is barely detectable in OE33, MKN28 and SW480 cells, likewise Bcl-x_L_ expression in H460 and H1299 cells. In most cell lines, the BH3-only protein Puma is barely detectable or only weakly expressed, with the exception of MKN28 with relatively strong Puma expression. The effect of Alectinib on the expression of Bcl-x_L_, Bcl-2 Bax, and Bak appears to be rather small overall. However, Puma expression is increased after Alectinib treatment in MDA-MB-231 and OE33 cells, but decreased in MKN28 and HCT116 cells (Supplemental Fig. 3).

VI. **Induction of apoptosis by Alectinib in a Bax/Bak double knockout line**

**Fig. 6:**
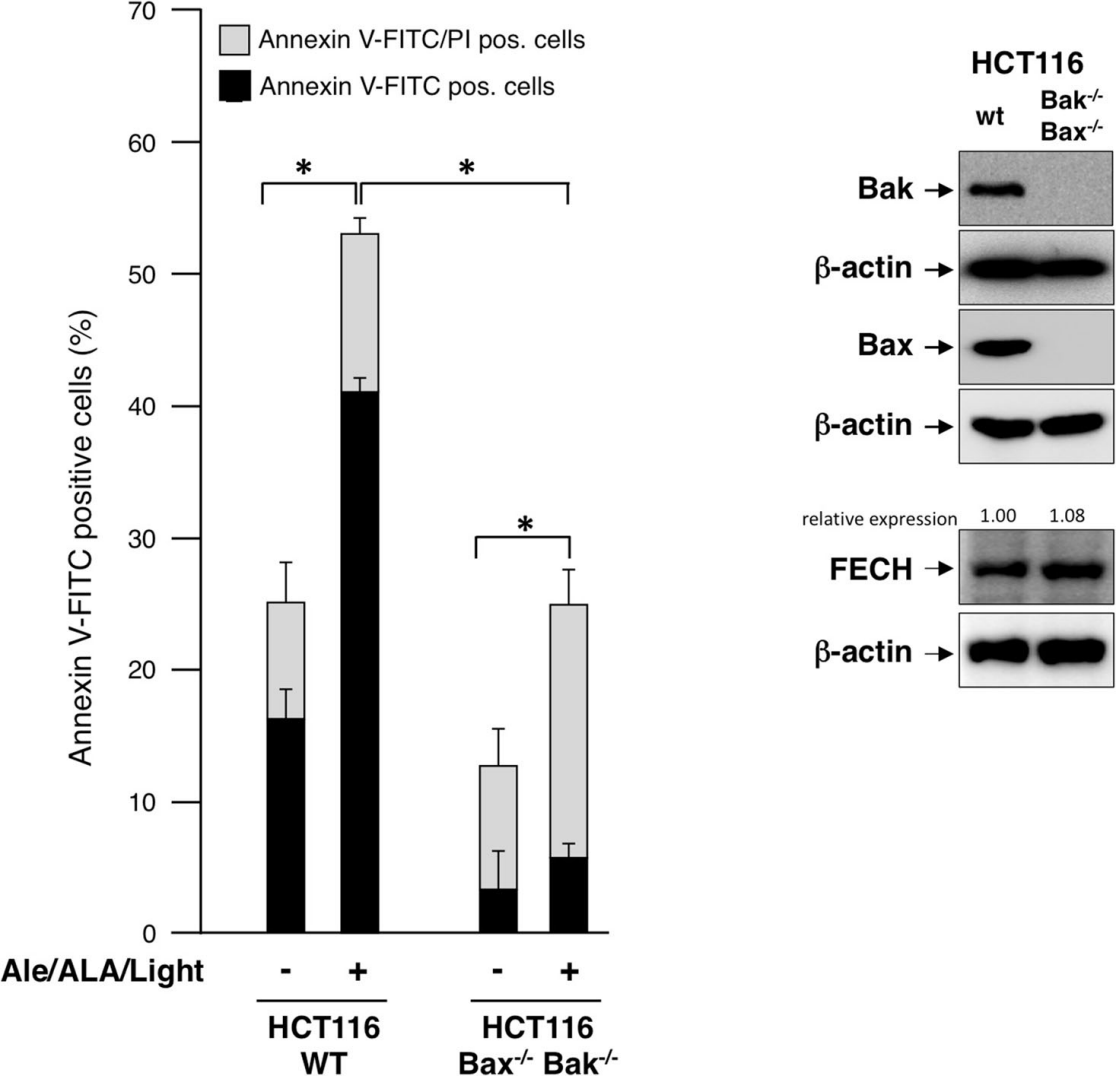
**Apoptosis Induction by Alectinib and light treatment in HCT116 Bax**^**−/−**^**/ Bak**^**−/−**^. HCT116 wild-type (WT) cells and isogenic double knockout subline HCT116-Bax^−/−^/Bak^−/−^ cells were treated with DMSO alone or 10 μM Alectinib/DMSO, 150 μM, and LED light. Combined treatment of cells with Alectinib and light induced cell death in both cell lines, but number of Annexin V-positive/PI-negative was significantly reduced and number of Annexin V-positive/PI-positive increased in the double knockout cells in comparison to HCT116 WT (Data show mean ± SD of three independent experiments; * *p <* 0.05). WB analysis (right side) confirmed loss of Bax and Bak expression in HCT116 double knock out cells and shows that FECH expression in HCT116-wt cells and isogenic HCT116-Bax^−/−^/Bak^−/−^ cells is comparable. Level of FECH expression was normalized to respective actin control and presented as relative protein expression level in relation to respective wild type control. (Analysis was performed twice. Both WB including quantification and indication of the mean value are shown in the supplemental Fig. 2)

The next question was whether cell death is induced by the intrinsic mitochondrial Bax/Bak dependent pathway or if other cell death signaling pathways are involved in cell death induction by the combined treatment. Therefore, HCT116 wild-type (wt) cells and the isogenic double knockout subline HCT116-Bax^−/−^/ Bak^−/−^ cells were treated as above (10 μM Alectinib, 150 μM 5-ALA for 3 h, and LED emitting light at a wavelength of 405 nm for one hour). Knockout of Bax and Bak was confirmed by WB analysis, showing loss of expression in HCT116-Bax^−/−^/ Bak^−/−^ cells (Fig. 6, right). Cells were stained with Annexin V-FITC/PI and analyzed by FACS. Combined treatment of cells with Alectinib, 5-ALA and light led to enhanced cell death in HCT116 wt cells. Especially the number of Annexin V-positive/PI-negative (early apoptotic) cells is strongly increased (Fig. 6), indicating induction of apoptosis upon treatment. Compared to wt cells the number of early apoptotic cells is strongly decreased in Bax/Bak deficient HCT116 cells. However, although Bax/Bak deficiency blocks induction of early apoptosis, the number of Annexin V-positive/PI-positive (late apoptotic/necrotic) cells increased from around 11% in the wt cell line to 19% in the HCT 116-Bax^−/−^/Bak^−/−^ cell line upon treatment (Fig. 6, right). Interestingly, the number of Annexin V-positive/PI- positive (late apoptotic/necrotic) cells shows a significant increase in HCT116 Bax^−/−^Bak^−/−^ upon treatment compared to untreated HCT116 Bax^−/−^Bak^−/−^ cells (Fig. 6, right columns). The differential sensitivity of wt and Bax/Bak deficient cells is not due to altered FECH expression as WB analysis reveals similar expression of FECH in both cell sublines (Fig. 6, right) demonstrating that sensitivity of cancer cells for cell death induction by Alectinib/light treatment essentially depends on the activity of various cell death signaling pathways.

**Fig. 7:**
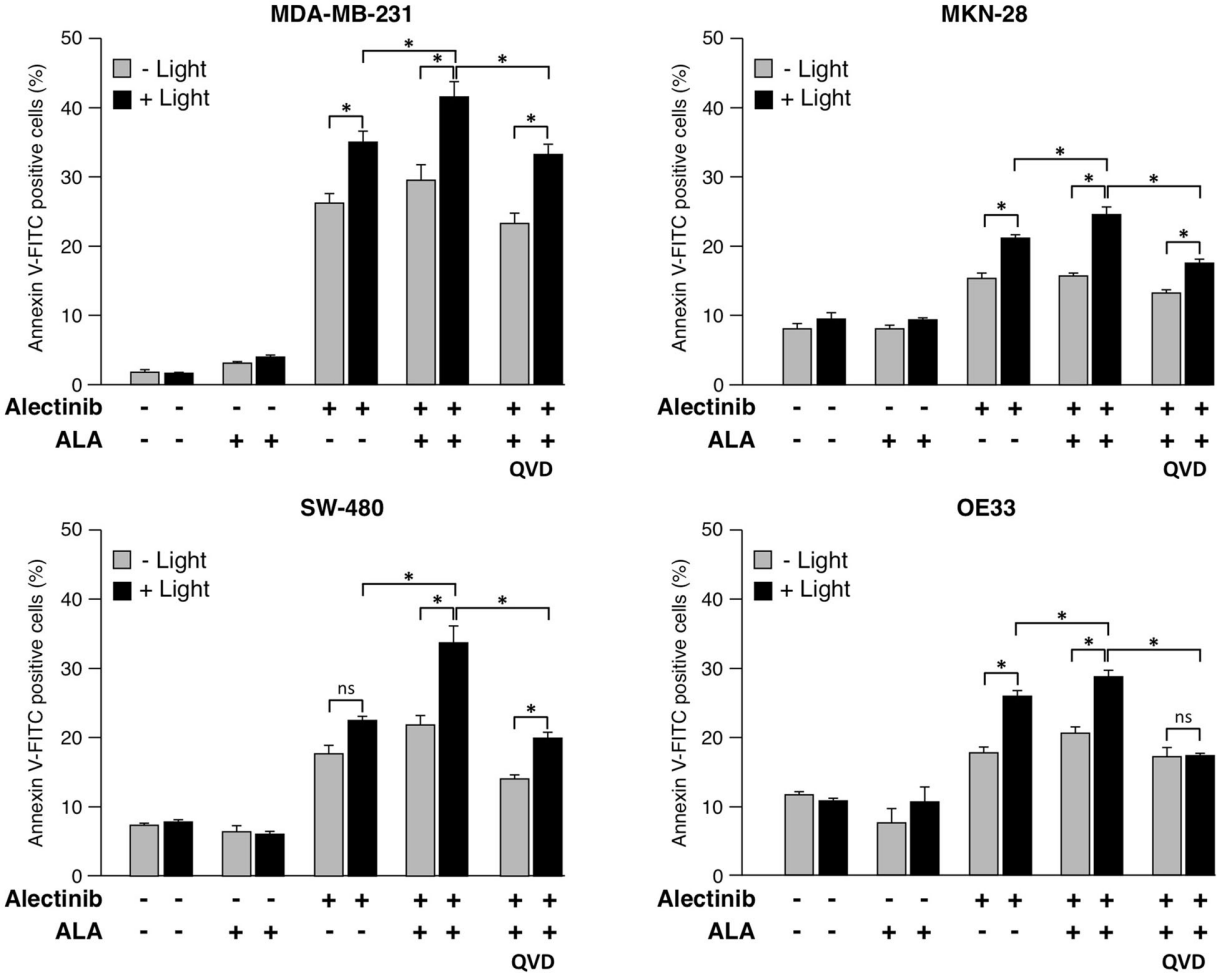
**Apoptosis Induction by Alectinib and light treatment upon caspase inhibition**. LED Light exposure enhances Alectinib-induced cell death in the cancer cell lines MDA-MB-231, MKN-28 and OE33 also in the absence of ALA. In all four cell lines (including SW-480) cell death induced by PDT after Alectinib treatment is further stimulated by 5-ALA. In order to investigate the role of caspases for Alectinib/PDT on a functional level, caspase activity was blocked by incubation of the cells with 10 μM of the cell-permeable, broad-spectrum caspase inhibitor Q-VD-Oph 2 h prior Alectinib/ALA/Light treatment. FACS analysis of treated cells revealed that caspase inhibition potently reduced the proportion of apoptotic cells in all cell lines. (Data show mean ± SD of three independent experiments; * *p <* 0.05)

In addition to the role of Bax and Bak we also analyzed whether caspases are involved in cell death induction upon combined Ale/ALA/Light treatment. Therefore, caspase activity was blocked by incubation of the tumor cell lines with the pan-caspase inhibitor Q-VD-OPh prior to the combined Ale/ALA/Light treatment. Flow cytometric analysis revealed that pretreatment of cells with the pan-caspase inhibitor Q-VD-OPh potently reduced the proportion of apoptotic cells in all cell lines tested (Fig. 7, right) indicating induction of apoptotic cell death upon combined treatment. Interestingly, neither Bax/Bak deficiency nor caspase inhibition completely inhibited cell death induction by the combined treatment, indicating that in addition to apoptosis alternative pathways are involved in the induction of cell death upon Ale/ALA/Light treatment.

## Discussion

A highly selective treatment for malignant cells is photodynamic treatment with 5-ALA. Beneficial effects of ALA-PDT have been demonstrated for various carcinomas, e.g. [12]. Recently, Hatakeyama et al. [13] showed that ALA-PDT using LED light is effective and useful for the treatment of colorectal carcinomas. Here, we were able to demonstrate that the efficiency of ALA-PDT could be enhanced by treatment of cancer cells with Alectinib, an orally available, highly selective, potent second-generation inhibitor of anaplastic lymphoma tyrosinkinase (ALK). Cancer cell lines of different origin such as the breast carcinoma line MDA-MB-231, esophageal adenocarcinoma OE33, gastric carcinoma MKN28, colorectal carcinoma SW480, and NSCLCs NCI-H460 and NCI-H1299 were examined. Combined treatment of cells with Alectinib and LED light led to enhanced cell death in each cell line (Fig. 5). Interestingly, since Alectinib promotes PpIX accumulation also in the ALK positive NSCLC tumor cell lines H1299 and H460, an additive treatment of NSCLC with ALA-PDT might enhance the beneficial therapeutic effect of Alectinib in these carcinomas. In contrast to the tumor cell lines, the rate of cell death is not increased by PDT with Alectinib treatment in non-tumorigenic human MCF-10A breast cells (Fig. 4). In agreement with the low sensitivity of non-tumorigenic MCF-10A cells to PDT with Alectinib shown here, a recent study has shown that non-tumorigenic human breast cells also are more resistant to PDT using methylene blue as photosensitizer (MB-PDT), whereas very aggressive triple-negative breast cancer cells (TNBCs) displayed high susceptibility to MB-PDT induced cell death [11]. Upon photooxidation MB disrupts lysosome integrity and induces lysosomal membrane permeabilization (LMP), which is accompanied by necroptosis or both necroptosis and ferroptosis in non-invasive and highly aggressive tumor cells, respectively. However, in non-tumorigenic human breast cell MB-PDT is accompanied by an antioxidant response and the enhanced ability of non-tumorigenic cells to deal with photo-oxidative damage confers resistance.

Another focus of our interest was to investigate whether cell death is induced by the intrinsic mitochondrial Bax/Bak dependent pathway or if other cell death signaling pathways are involved in cell death induction by the combined treatment. Inhibition of the intrinsic apoptosis pathway by Bax/Bak deletion only partially inhibits cell death induced by Ale/ALA/Light treatment. Likewise, inhibition of caspases by the pan-caspase inhibitor Q-VD-OPh only provides partial protection against the treatment, indicating that alternative caspase-independent mechanisms are involved in the induction of cell death. In line with these results, it has been shown, that PDT can kill cancer cells by the efficient induction of apoptotic as well as of non-apoptotic cell death pathways including necrosis and necroptosis, depending on the photosensitizer, PDT protocol, and tumor type [14–17]. These different forms of cell death are not mutually exclusive. Furthermore, induction of autophagy in photosensitized cells is a common phenomenon of PDT [18]. In apoptosis-deficient murine embryonic fibroblasts lacking Bax and Bak PDT stimulates a form of caspase-independent cell death, with a necrotic morphotype, for which aberrant autophagy stimulation is required [19]. However, the role of autophagy as a factor in PDT-induced cell death is not yet clear, as autophagy can also serve as a protective mechanism [17, 20]. An interesting paper published recently by Dos Santos et al. [11] showed that in breast cancer cells lysosomal membrane permeabilization is a common event upon PDT which is accompanied by an antioxidant response in non-tumorigenic cells, necroptosis in non-invasive tumor cells, and both necroptosis and ferroptosis in highly aggressive triple-negative tumor cells.

As mentioned above, Alectinib demonstrates a favorable safety profile and clinically meaningful response in patients with ALK-positive metastatic NSCLC [8] and was granted accelerated approval by the FDA. Our data suggest that an additive treatment of NSCLC with ALA-PDT might even enhance the beneficial therapeutic effect of Alectinib in these carcinomas. On the other hand, Alectinib has some side effects such as hepatic toxicity (see e.g. Rxlist, https://www.rxlist.com). This has to be checked very carefully in the clinical setting. In the future there might be alternative PDT-treatments with less side effects, however still the majority of current on-going trials are using photosensitizers that have already approved for clinical use, such as ALA [21]. As described here, the results of ALA-PDT could be improved by Alectinib. In the case of patients suffering from metastasized gastrointestinal carcinomas there are only very few treatment options. Therefore, in these cases the risk of side effects of ALA-Alectinib-PDT can be accepted.

Peritoneal dissemination represents a devastating form of colorectal, gastric, pancreatic or ovarian cancer progression with a dismal prognosis. Colorectal cancer is the third common cancer, of which the peritoneum is the second most common metastatic site [22] [23]. Peritoneal dissemination is identified in nearly 10% of colorectal cancer patients when initially diagnosed, and in 20–50% of patients with recurrent disease [22]. For gastric cancer, the 5-year survival rate of patients with peritoneal dissemination is only 2% [24]. Diagnosing minimal peritoneal dissemination improves the prognosis because the patients undergo chemotherapy treatment earlier. Kondo et al. [25] observed better diagnostic accuracy using 5-ALA photodynamic diagnosis compared to conventional laparoscopy in patients with colorectal cancer. According to their investigations, 5-ALA photodynamic diagnosis is a promising candidate for diagnosing peritoneal dissemination in colorectal cancer. Recent papers discusses the perspectives of photodynamic diagnosis for peritoneal dissemination [26] [27].

## Conclusions

Photodynamic treatment of tumor cell lines of different origin in combination with Alectinib increased protoporphyrin-IX specific fluorescence and concomitantly cell death. Thus, the usage of Alectinib could be another step for enhancing the effectiveness of photodynamic therapy. Since Alectinib promotes PpIX accumulation also in the ALK positive NSCLC tumor cell lines H1299 and H460, an additive treatment of NSCLC with ALA-PDT might enhance the beneficial therapeutic effect of Alectinib in these carcinomas. Moreover, further experiments in animal models with peritoneal metastases of gastrointestinal carcinomas will show whether a combination of an ALA based photodynamic therapy with Alectinib treatment could be a new strategy for diagnosis or even treatment of peritoneal disseminated carcinomas.

## Supplementary Information

**Additional file 1: Supplemental Fig. 1.** Increased fluorescence-intensity upon Alectinib treatment. Mean fluorescence-intensity of NSCLC cells H460, H1299 and MDA-MB-231 breast cancer cells was increased upon Alectinib treatment while Crizotinib was not effective. Cells were treated as described in Fig. 1. Fluorescence images were assessed using Axiovert 200 M Inverted Fluorescence Microscope and mean fluorescence-intensity analyzed by Image 1.53a software. (mean ± standard deviation of three experiments; * *p* < 0.05).

**Additional file 2: Supplemental Fig. 2.** Quantification of FECH expression. *FECH Western Blots were quantified by use of the* ImageLab software 6 (Bio-Rad). Level of FECH expression, was normalized to the respective actin control and presented as the relative expression level in relation to respective wild type control. Analysis of FECH expression by WB analysis was performed twice in independent experiments. The upper blots are identical to the respective blots from Figs. 5 and 6.

**Additional file 3: Supplemental Fig. 3.** Expression levels of key pro- and anti-apoptotic Bcl-2 family members. Cell lines strongly express Bak and Bax, with the strongest Bax expression seen in MKN28 and SW480 cells. Expression of anti-apoptotic Bcl-2 and Bcl-x_L_, varies between cell lines. Puma is barely detectable or only weakly expressed, with the exception of MKN28 with relatively strong Puma expression. The effect of Alectinib on expression appears to be rather small overall. However, Puma expression is increased after Alectinib treatment in MDA-MB-231 and OE33 cells, but decreased in MKN28 and HCT116 cells (quantified by ImageLab software 6 (Bio-Rad), − not calculated due to low expression).

**Additional file 4: Supplemental Fig. 4.** Intensity of LED light source. Measured intensity (power per area) vs. supply current for the 405 nm LED lamp. Parameter is the distance between lamp and illuminated area. If the is lamp operated at 25 mA and the distance between LED and sample is 25 mm, intensity is 4 mW/cm^2^.

## Data Availability

See Methods section.

## Abbreviations

5-ALA: 5-Aminolevulinic acid
ALA-PDT: Aminolevulinic acid-mediated photodynamic therapy
ALK: Anaplastic lymphoma tyrosinkinase
FECH: Ferrochelatase (EC 4.99.1.1)
FITC: Fluorescein isothiocyanate
LED: Light Emitting Diode
NSCLC: Non-small-cell lung carcinoma
PI: Propidium iodide
PpIX: Protoporphyrin-IX
ROS: Reactive oxygen species (ROS)
WB analysis: Western blot analysis
Wt: Wild-type cells
TKI: Multiple tyrosine kinase inhibitor
