# Supplementary material for: Alectinib treatment improves photodynamic therapy in cancer cell lines of different origin
Source: BMC Cancer. 2021 Aug 30;21:971. doi: 10.1186/s12885-021-08667-x (PMC8404354; doi:10.1186/s12885-021-08667-x)

Supplemental Fig 3

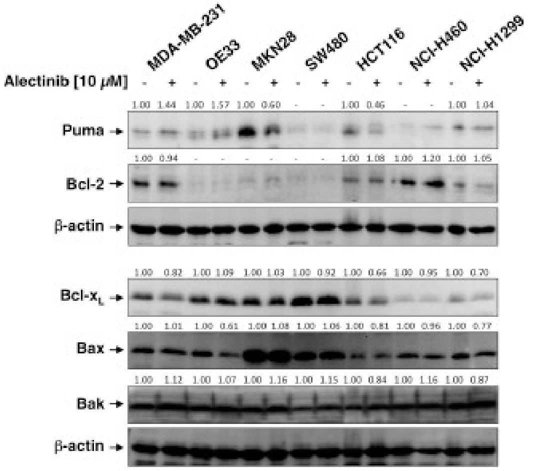

Supplement: Supplementary file 3 [file 12885_2021_8667_MOESM3_ESM.docx]
